# Educational experiences associated with cold- and hot-run practical training on the handling of unsealed radioactive sources by radiological technologist students

**DOI:** 10.1186/s12909-026-09594-w

**Published:** 2026-06-05

**Authors:** Akihiro Kakimoto, Shota Watanabe, Yutaka Otaka, Yui Murayama, Mitsuki Yoshimoto, Daisuke Fujise, Shin Hasegawa

**Affiliations:** 1 Department of Radiological Sciences, Faculty of Medical Science Technology, Morinomiya University of Medical Sciences, 1-26-16 Nankokita, Suminoe-ku, Osaka-shi, Osaka, 559-8611 Japan; 2 Department of Biofunctional Imaging, Preeminent Medical Photonics Education and Research Center, Hamamatsu University School of Medicine, Hamamatsu-shi, Shizuoka, Japan; 3 Department of Information Technology and Management, IT Planning Section, National Institutes for Quantum Science and Technology, Chiba-shi, Chiba, Japan

**Keywords:** Education, Radiological technologist students, Radiological protection, Radiopharmaceutical aliquoting

## Abstract

**Background:**

This study aimed to evaluate students’ subjective educational experiences related to cold- and hot-run practical training on the handling of unsealed radioactive sources by radiological technologists, using quantitative and qualitative methods. To the best of our knowledge, few studies have provided a structured quantitative comparison of perceived safety-related learning experiences between cold- and hot-run training in nuclear medicine.

**Methods:**

In total, 143 students participated in this study. A five-point Likert scale questionnaire was used to evaluate primary and secondary outcomes related to cold- and hot-run training, and open-ended responses were analyzed using a qualitative descriptive methodology.

**Results:**

The quantitative results showed positive self-reported educational experiences for both procedures; however, hot-run training yielded significantly higher scores in safety awareness and learning satisfaction, including safety-related secondary outcomes, suggesting distinct perceived educational characteristics. The fourth-year students demonstrated lower self-assessment scores than the third-year students across all items. The qualitative findings highlighted the tension, clinical realism, and heightened perceptions of safety awareness associated with hot-run experiences.

**Conclusions:**

These results suggest that combined cold- and hot-run training is perceived as educationally valuable; however, additional educational strategies may be required to support sustained self-confidence and perceived understanding over time.

**Supplementary Information:**

The online version contains supplementary material available at 10.1186/s12909-026-09594-w.

## Background

In recent years, healthcare system reforms in Japan have promoted task shifting and sharing to address physicians’ workloads, resulting in the increased involvement of radiological technologists in the preparation and handling of radiopharmaceuticals [1]. Consequently, safe and appropriate handling of unsealed radioactive sources has become a core competency in nuclear medicine and a key professional responsibility from the perspective of radiation protection. Guidelines on the handling of unsealed radioactive sources have been provided both internationally, such as by the World Health Organization [2], and domestically by relevant Japanese academic societies, which continue to update their recommendations. These guidelines emphasize not only the technical aspects of practical tasks but also the importance of institution-specific practical education, training programs, and safety management systems. However, legal regulations, facility requirements, and radiation exposure management constraints limit opportunities for hands-on training, leading to substantial variability in the content and frequency of practical education among training institutions.

In this context, it is important to evaluate the educational value of existing practical training methods. Practical training typically involves both simulated exercises (cold runs) and actual handling (hot runs). However, evidence comparing perceived educational characteristics and safety-related learning experiences remains limited, and few studies have provided a structured, quantitative comparison of safety-related learning experiences between cold- and hot-run training in nuclear medicine.

Thus, this study aimed to compare the subjective educational experiences associated with cold- and hot-run practical training among technologist students, focusing on procedural understanding, safety awareness, learning satisfaction, and self-confidence related to handling unsealed radioactive sources.

To achieve this aim, we used a mixed-methods approach combining quantitative questionnaire-based evaluation and qualitative analysis of open-ended responses. Additionally, alongside the structured quantitative assessment, a qualitative analysis was performed to examine the experiential aspects of learning, including clinical realism, tension, and perceptions regarding radiation safety, which may not be adequately captured through quantitative measures alone. By integrating the quantitative and qualitative findings, this study sought to provide a more comprehensive understanding of the educational characteristics associated with cold- and hot-run nuclear medicine training.

## Methods

### Ethics statement

This study was reviewed and approved by the university’s institutional ethics committee (approval number: 2025-069). All eligible students received an explanation of the study purpose, data collection procedures, and data handling, and written informed consent was obtained. Participation was voluntary, and all responses were anonymized, securely managed, and reported in a manner that prevented individual identification. Participants were informed that study participation would not affect their academic evaluation, and data collection was conducted after the release of their final grades for the relevant academic term. Study information sheets, consent forms, and withdrawal forms were distributed in a paper format, and consent was confirmed using Microsoft Forms. The survey was conducted between October 1 and October 31, 2025.

### Participants

**Fig. 1:**
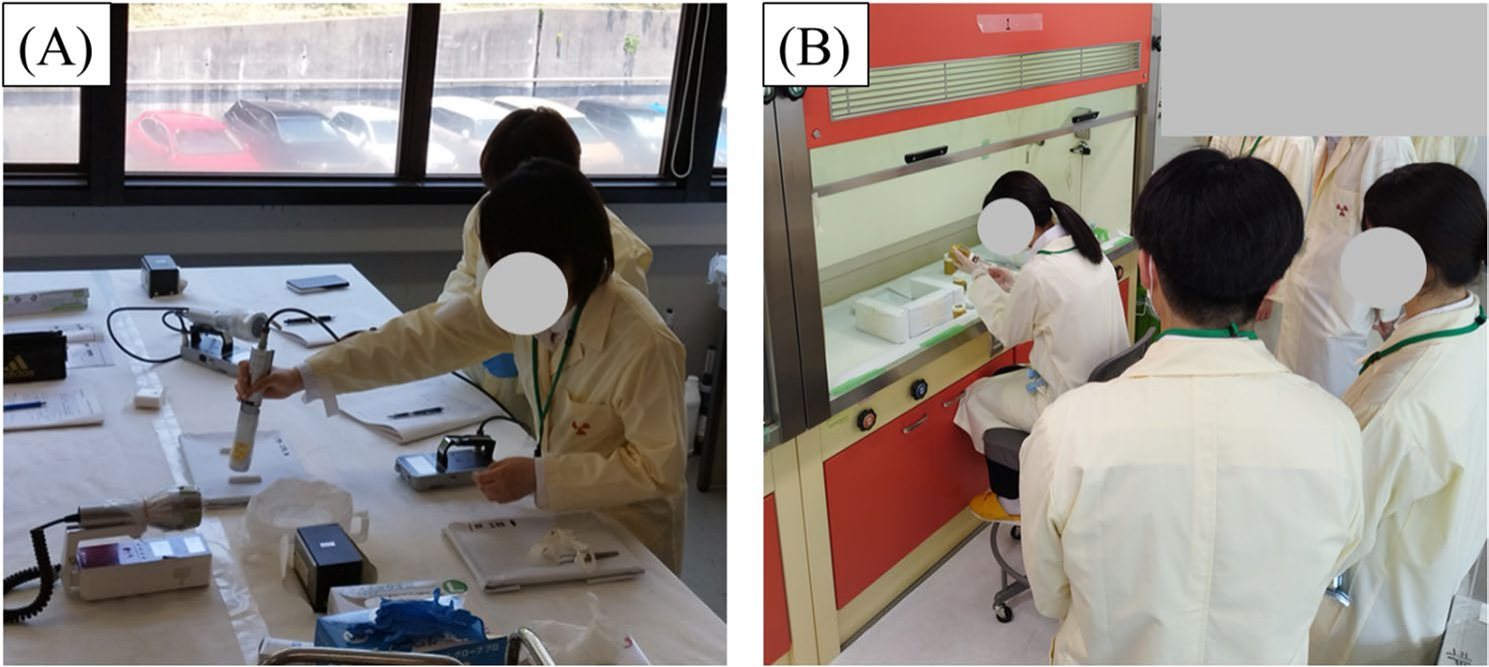
Practical training involving the handling of unsealed radioactive sources. **A** Measurement of surface contamination density using a GM survey meter. **B** Aliquoting operation using a ^99^Mo–^99m^Tc generator

**Fig. 2:**
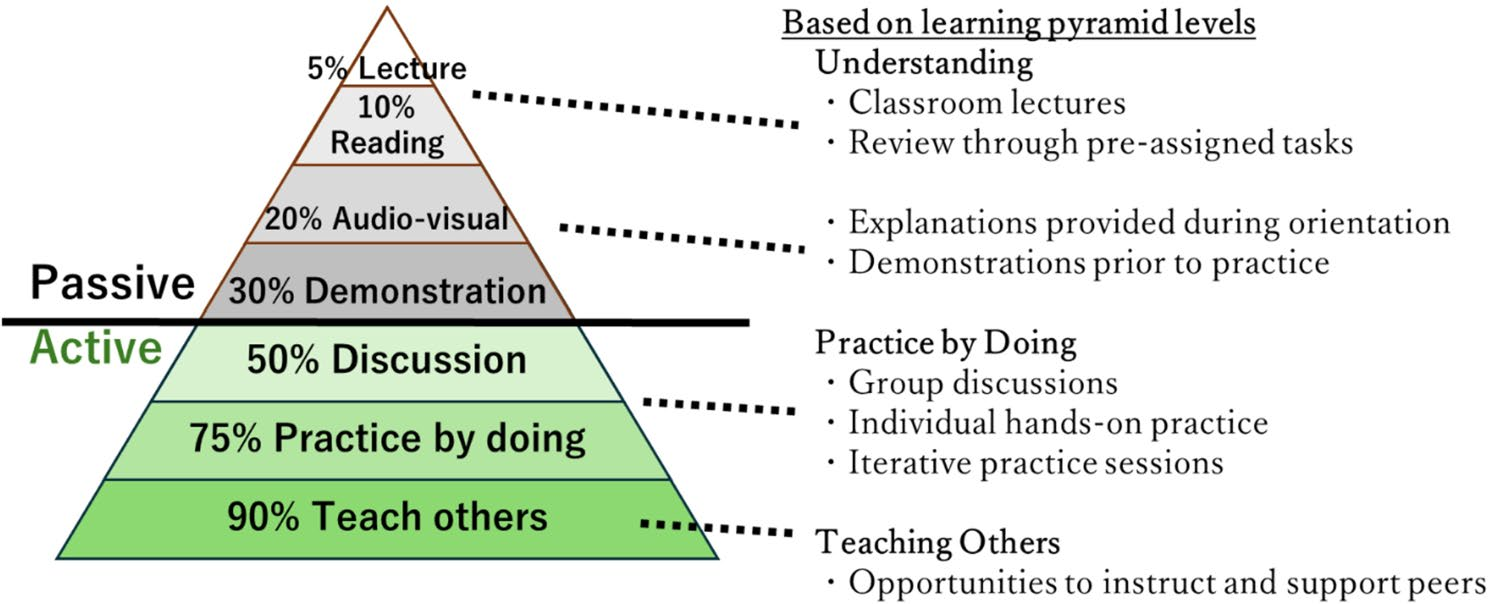
Practical training style based on the learning pyramid. Stepwise practical training design based on the learning pyramid. Students first engaged in passive learning through lectures, pre-class assignments, and instructional videos or demonstrations prepared by instructors. This was followed by hands-on practical training in small groups. Finally, students reflected on their own procedures through active group discussions and written reports, aiming to support progressive learning experiences from knowledge acquisition to practical performance and explanations to others

The study included 143 students enrolled in a radiological technologist program, comprising 80 third-year and 63 fourth-year students. All participants completed curriculum-defined coursework in radiation safety management and nuclear medicine techniques, as specified in the national guidelines for radiological technologist training. As part of a nuclear medicine practical course, students conducted surface contamination measurements and reagent dispensing using vials and syringes, completing 4.5 h and 1.5 h of cold- and hot-run training, respectively (Fig. 1). Practical sessions were designed based on the learning pyramid [3], combining lectures, demonstrations, small-group hands-on practice, and reflective exercises (Fig. 2). Questionnaire data were collected approximately 3 months after training for third-year students and 15 months after training for fourth-year students. Therefore, comparisons between year groups reflect differences between independent student cohorts assessed at different post-training intervals rather than longitudinal within-subject changes.

### Questionnaire development and preliminary support for validity

In this study, the questionnaire used was developed by the authors to evaluate students’ subjective educational experiences related to handling unsealed radioactive sources. The questionnaire items were developed by three university faculty members who were licensed radiological technologists involved in practical nuclear medicine education.

The questionnaire structure was designed based on the learning pyramid concept, with items arranged hierarchically from procedural understanding and safety awareness to confidence in independent practice and the ability to explain procedures to others. Items A1–A3, B1–B3, and C1–C3 focused on understanding and awareness, whereas items A4–A5, B4–B5, and C4–C5 evaluated perceived confidence in independent performance and the ability to explain procedures to others. Items D1–D3 and E1–E3 assessed the overall satisfaction and self-confidence related to practical training.

To evaluate content validity and clarity, pilot testing was conducted with 10 students and six faculty members who were not involved in questionnaire development. Internal consistency reliability was evaluated using the Cronbach’s alpha coefficient [4] calculated from the pilot test responses. The questionnaire demonstrated excellent internal consistency (Cronbach’s α = 0.91). Cronbach’s alpha values for domains A–E were 0.86, 0.68, 0.90, 0.70, and 0.73, respectively, indicating acceptable internal consistency.

Exploratory factor analysis (EFA) was conducted using responses from both cold- and hot-run datasets to preliminarily assess construct validity. Factor extraction was performed using principal axis factoring with Promax rotation [5]. Sampling adequacy was evaluated using the Kaiser–Meyer–Olkin (KMO) measure [6] and Bartlett’s test of sphericity [7].

### Quantitative evaluation

A quantitative evaluation was conducted using a self-administered questionnaire based on a five-point Likert scale (1 = strongly disagree, 2 = disagree, 3 = neutral, 4 = agree, and 5 = strongly agree) [8]. The primary outcome items were defined as overarching learning domains, including operational understanding, safety awareness, and overall satisfaction with practical training, and were calculated as the mean scores of the corresponding secondary outcome items (Table 1). The secondary outcome items evaluated specific aspects of procedural understanding, confidence, safety awareness, and self-assessed confidence in handling unsealed radioactive sources. These items were structured hierarchically to reflect progressive learning levels, ranging from understanding and awareness to confidence in independent performance and the ability to explain procedures to others. The authors developed the questionnaire to assess students’ self-perceived attainment of predefined learning milestones, including knowledge retention, independent practice, and teaching ability. The full version of the questionnaire, including response options, is provided in Supplementary File 1.

**Table 1:** Questionnaire items

Primary outcomes	Secondary outcomes
A. Procedural understanding & confidence (Surface contamination measurement)	A1. Understood the procedures for direct measurement
	A2. Understood the procedures for indirect measurement
	A3. Understood the calculation methods and criteria for removal from controlled areas
	A4. Confident in performing all three procedures
	A5. Confident in explaining all three procedures to others
B. Procedural understanding & confidence (Syringe/vial handling, ^99m^Tc generator operation)	B1. Understood the operational procedures for the generator
	B2. Understood the operational procedures for radiopharmaceutical dispensing
	B3. Understood calculations for generator, radioactive decay, and decay correction
	B4. Confident in performing all three procedures independently
	B5. Confident in explaining all three procedures to others
C. Safety awareness & radiation protection	C1. Understood safety procedures for handling unsealed radioactive sources
	C2. Learned practical methods to reduce radiation exposure
	C3. Felt that safety awareness increased through this training/operation
	C4. Confident in performing all three items independently
	C5. Confident in explaining all three items to others
D. Learning satisfaction	D1. Felt that the learning effects through the training and procedures were sufficient
	D2. Satisfied with the content and operations of this training
	D3. Felt that this training/procedure will be useful for future work
E. Self-confidence & self-assessment	E1. Felt confident in the knowledge and skills gained from this training/procedure
	E2. Felt capable of performing the same procedures independently
	E3. Felt able to perform the procedures correctly in the future based on this experience
Open-ended response	-

Primary outcome domains were defined as composite scores calculated from the mean values of the corresponding secondary outcome items. The secondary outcome items assessed specific aspects of procedural understanding, confidence, safety awareness, and self-assessed confidence in handling unsealed radioactive sources. Items A–C consisted of five hierarchical levels: understanding and awareness (A1–A3, B1–B3, and C1–C3), confidence in independent performance (A4, B4, and C4), and confidence in explaining the procedures to others (A5, B5, and C5). All items were rated on a five-point Likert scale. Open-ended responses were included in the qualitative analysis

Cold-run training was conducted in a simulated environment without radioactive materials, whereas hot-run training involved handling unsealed radioactive sources in a radiation-controlled area. The mean scores for both the primary and secondary outcome items were compared between the cold- and hot-run conditions to evaluate the differences in perceived educational experiences.

Third- and fourth-year students were compared to examine the between-cohort differences in their subjective evaluations at different post-training intervals. Statistical analyses were performed using independent two-sample t-tests, and statistical significance was set at *p* < 0.05. Bonferroni correction was employed to adjust for multiple comparisons across outcome items. All quantitative analyses were performed using Microsoft Excel (Microsoft Corporation, Redmond, WA, USA).

### Qualitative analysis

Open-ended responses were analyzed using a qualitative descriptive approach based on Kawakita’s KJ Method, a systematic technique for organizing and interpreting qualitative data, as described by Scupin [9], and complemented by established qualitative analysis frameworks [10–12]. Two independent researchers who were neither involved in educational instruction nor participated in the training conducted the analysis to ensure analytical neutrality.

First, all open-ended responses were read repeatedly to familiarize the participants with the data and identify meaningful statements related to learning experiences, operational understanding, and safety awareness. These statements were then divided into discrete meaning units. Meaning units were then grouped according to similarities in content, and descriptive labels were assigned to each group. Following the principles of the KJ Method, related groups were iteratively compared and integrated into higher-order categories from which overarching themes representing students’ subjective perceptions of training were derived. This analytical process was conducted to structurally extract learners’ subjective experiences and perceptions of educational experiences that could not be fully captured by a Likert scale-based quantitative evaluation.

## Results

### Quantitative findings

**Fig. 3:**
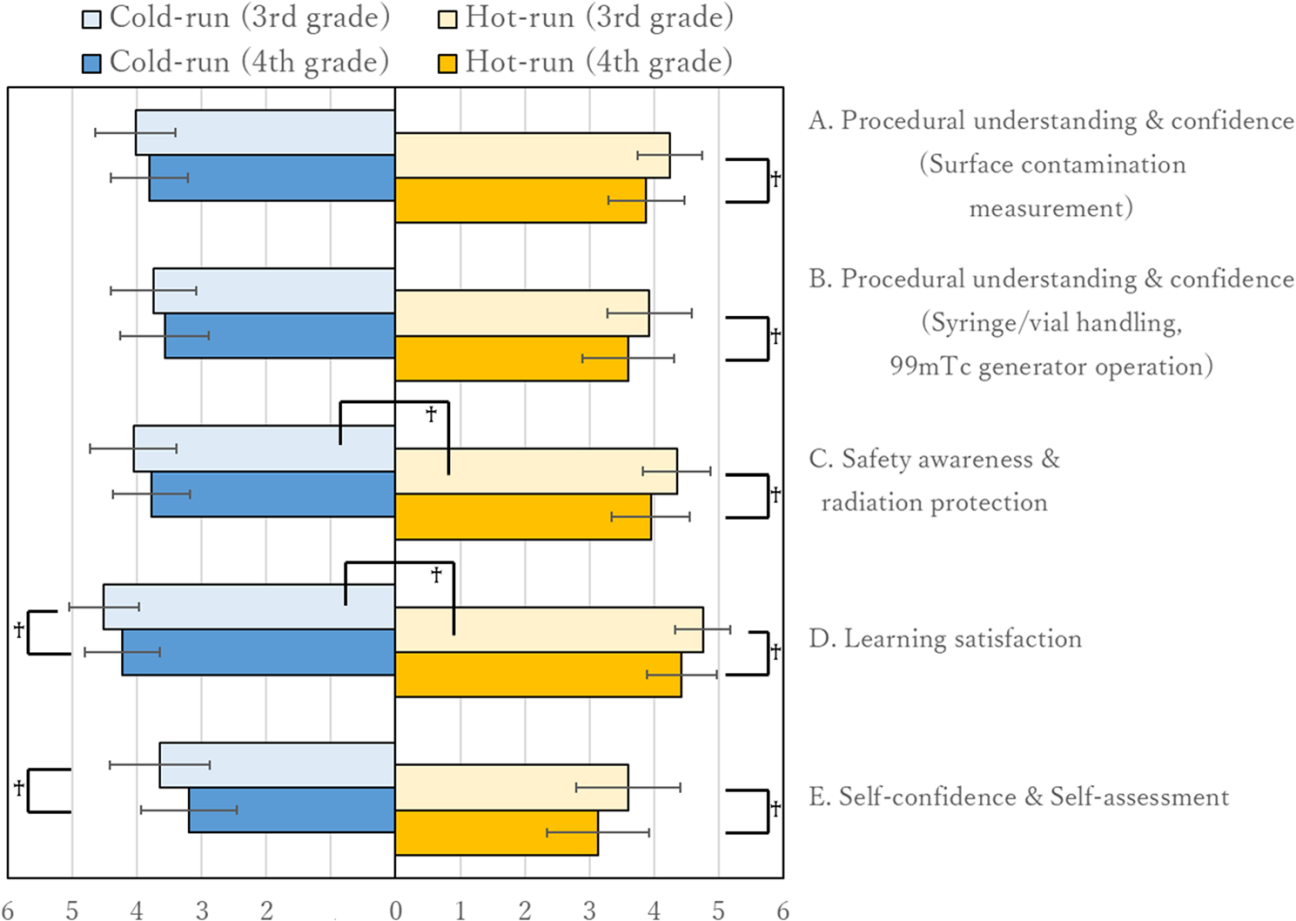
Comparison of primary outcome scores between cold- and hot-run training. The mean primary outcome scores (± standard deviation) for cold- and hot-run training are shown for third- and fourth-year students. The primary outcomes included (**A**) self-assessed procedural understanding and confidence related to surface contamination measurement; (**B**) self-assessed procedural understanding and confidence related to syringe/vial handling and ^99m^Tc generator operation; (**C**) safety awareness and radiation protection; (**D**) learning satisfaction; and (**E**) self-confidence and self-assessment. Scores were assessed using a five-point Likert scale (1 = strongly disagree to 5 = strongly agree). Brackets indicate comparisons between cold- and hot-run conditions within the same grade. ☨ indicates a statistically significant difference (*p* < 0.05, Bonferroni corrected)

Both cold- and hot-run trainings showed high mean scores across all primary and secondary outcome items (Tables 2 and 3). When comparing training modalities, hot-run procedures were associated with significantly higher scores on the primary outcome items related to safety awareness and learning satisfaction. Additionally, the secondary outcome items associated with perceptions related to safety-related practices and radiation protection showed significantly higher ratings under hot-run conditions (Fig. 3).

**Table 2:** Average scores of primary outcome measures for cold- and hot-run training

Primary outcomes	Third grade			Fourth grade		
	Cold-run	Hot-run	Significant difference (cold and hot-run)	Cold-run	Hot-run	Significant difference (cold and hot-run)
A. Procedural understanding & confidence (Surface contamination measurement)	4.03 ± 0.62	4.25 ± 0.50	n.s.	3.81 ± 0.59	3.88 ± 0.59	n.s.
B. Procedural understanding & confidence (Syringe/vial handling, ^99^mTc generator operation)	3.75 ± 0.65	3.93 ± 0.66	n.s.	3.57 ± 0.68	3.60 ± 0.52	n.s.
C. Safety awareness and radiation protection	4.06 ± 0.67	4.35 ± 0.52	☨	3.78 ± 0.59	3.95 ± 0.61	n.s.
D. Learning Satisfaction	4.51 ± 0.54	4.75 ± 0.43	☨	4.23 ± 0.58	4.42 ± 0.54	n.s.
E. Self-confidence & self-assessment	3.65 ± 0.78	3.61 ± 0.81	n.s.	3.20 ± 0.74	3.14 ± 0.79	n.s.

The average scores (mean ± standard deviation) of the primary outcome measures assessed using a five-point Likert scale (1 = strongly disagree, 2 = disagree, 3 = neutral, 4 = agree, and 5 = strongly agree) are shown for third- and fourth-year students. Primary outcome scores were calculated from the mean values of the corresponding secondary outcome items within each domain. Comparisons between cold- and hot-run conditions were conducted separately for each grade. ☨ indicates a statistically significant difference between the cold- and hot-run conditions (*p* < 0.05, Bonferroni corrected), *n.s*. indicates no significant difference

**Table 3:** Scores of secondary outcome items on a 5-point Likert scale

No.	3rd grade			4th grade			SD3	SD4
	Cold-run	Hot-run	SD1	Cold-run	Hot–run	SD2		
A1.	4.28 ± 0.75	4.65 ± 0.58	☨	4.25 ± 0.65	4.44 ± 0.62	n.s.	n.s.	n.s.
A2.	4.21 ± 0.74	4.66 ± 0.50	☨	4.25 ± 0.65	4.41 ± 0.61	n.s.	n.s.	n.s.
A3.	4.30 ± 0.72	4.54 ± 0.57	n.s.	4.00 ± 0.80	4.19 ± 0.80	n.s.	n.s.	n.s.
A4.	3.88 ± 0.80	3.78 ± 0.78	n.s.	3.44 ± 0.98	3.22 ± 0.85	n.s.	n.s.	☨
A5.	3.46 ± 0.81	3.60 ± 0.77	n.s.	3.10 ± 0.82	3.13 ± 0.83	n.s.	n.s.	☨
B1.	3.70 ± 0.91	4.04 ± 0.88	n.s.	3.70 ± 0.89	3.78 ± 0.85	n.s.	n.s.	n.s.
B2.	4.23 ± 0.66	4.49 ± 0.64	n.s.	4.10 ± 0.69	4.21 ± 0.70	n.s.	n.s.	n.s.
B3.	3.85 ± 0.83	4.05 ± 0.88	n.s.	3.60 ± 0.87	3.70 ± 0.93	n.s.	n.s.	n.s.
B4.	3.58 ± 0.81	3.56 ± 0.84	n.s.	3.30 ± 0.85	3.24 ± 0.91	n.s.	n.s.	n.s.
B5.	3.38 ± 0.83	3.50 ± 0.84	n.s.	3.14 ± 0.82	3.10 ± 0.89	n.s.	n.s.	n.s.
C1.	4.06 ± 0.79	4.43 ± 0.65	☨	3.89 ± 0.76	3.97 ± 0.76	n.s.	n.s.	☨
C2.	4.16 ± 0.86	4.60 ± 0.65	☨	4.03 ± 0.72	4.30 ± 0.66	n.s.	n.s.	n.s.
C3.	4.31 ± 0.82	4.81 ± 0.48	☨	4.11 ± 0.72	4.54 ± 0.64	☨	n.s.	n.s.
C4.	4.00 ± 0.78	4.09 ± 0.78	n.s.	3.54 ± 0.80	3.60 ± 0.83	n.s.	☨	☨
C5.	3.74 ± 0.76	3.84 ± 0.75	n.s.	3.33 ± 0.80	3.33 ± 0.84	n.s.	n.s.	☨
D1.	4.46 ± 0.59	4.74 ± 0.55	n.s.	4.14 ± 0.62	4.35 ± 0.60	n.s.	☨	☨
D2.	4.51 ± 0.64	4.73 ± 0.55	n.s.	4.22 ± 0.79	4.44 ± 0.67	n.s.	n.s.	n.s.
D3.	4.56 ± 0.59	4.80 ± 0.43	n.s.	4.32 ± 0.64	4.48 ± 0.62	n.s.	n.s.	☨
E1.	3.84 ± 0.80	3.88 ± 0.86	n.s.	3.40 ± 0.83	3.48 ± 0.82	n.s.	☨	n.s.
E2.	3.44 ± 0.95	3.35 ± 0.98	n.s.	3.05 ± 0.94	2.89 ± 0.94	n.s.	n.s.	n.s.
E3.	3.68 ± 0.85	3.60 ± 0.84	n.s.	3.14 ± 0.88	3.05 ± 0.96	n.s.	☨	☨

Values are presented as mean ± standard deviation. SD1 indicates a significant difference between cold- and hot-run training among third-year students. SD2 indicates a significant difference between cold- and hot-run training in fourth-year students. SD3 indicates a significant difference between third- and fourth-year students under the cold-run conditions. SD4 indicates a significant difference between third- and fourth-year students in the hot-run conditions. ☨ indicates a statistically significant difference (*p* < 0.05, Bonferroni correction), *n.s.* indicates no significant difference

Exploratory factor analysis demonstrated acceptable suitability for factor analysis in both cold- and hot-run datasets (cold-run: KMO = 0.85; hot-run: KMO = 0.81; Bartlett’s test of sphericity, *p* < 0.001 for both). Four factors with eigenvalues greater than 1.0 were identified in both datasets. The extracted factors generally correspond to the conceptual domains of procedural understanding, safety awareness, learning satisfaction, and self-confidence, thus providing preliminary support for construct validity of the questionnaire.

Comparisons between third- and fourth-year students indicated lower scores across most outcome items for both the cold- and hot-run training programs. Although fourth-year students continued to demonstrate relatively high mean scores (approximately 3.7–4.0), lower ratings were consistently observed compared to third-year students across most outcome items, reflecting differences between the cohorts assessed at different post-training intervals.

### Qualitative findings

In total, 143 open-ended responses were analyzed. Responses with similar meanings or contents were categorized into five themes. When a single response contained multiple themes, each theme was counted separately. The results are summarized in Table 4.

**Table 4:** Results of qualitative descriptive analysis of open-ended responses in cold- and hot-run practical training

Theme	3rd grade (*n* = 80)	4th grade (*n* = 63)	Total (*n* = 143)
1. Tension/nervousness	32 (40.0%)	24 (38.1%)	56 (39.2%)
2. Awareness of safety/radiation risk	40 (50.0%)	32 (50.8%)	72 (50.3%)
3. Cold-run supporting hot-run	27 (33.8%)	39 (61.9%)	66 (46.2%)
4. Learning retention/impression	43 (53.8%)	29 (46.0%)	72 (50.3%)
5. Relevance to future clinical practice	23 (28.8%)	7 (11.1%)	30 (21.0%)

Values represent the number of responses, with percentages in parentheses. In total, 143 open-ended responses were analyzed. When a single response contained multiple themes, it was considered to belong to more than one category. Percentages were calculated based on the total number of respondents in each grade

#### Tension and clinical realism

Descriptions related to tension, fear, or perceived risk during hot-run training accounted for 39.2% of the responses. By academic year, this theme was identified in 32 (40.0%) third- and 24 (38.1%) fourth-year students. Typical comments included statements such as “I felt tension that I could not experience during cold-run training” and “handling real radioactive materials made the situation feel real.” These responses were associated with a greater sense of clinical realism, which the students reported less frequently during lectures or simulated exercises alone.

#### Safety management and radiation risk awareness

Statements related to safety management and radiation exposure risk were the most frequently reported themes, accounting for 50.3% (72 responses). The proportions were similar between the third- (40 responses, 50.0%) and fourth-year students (32 responses, 50.8%). Many students described a heightened awareness of minimizing exposure, preventing contamination, and shortening working duration. These comments reflect the students’ attentiveness to radiation protection during hot-run procedures.

#### Supportive role of cold-run training

Comments describing the utility of cold-run training were identified in 46.2% of the responses (66 responses). By academic year, this theme appeared in 27 (33.8%) and 39 (61.9%) responses from third- and fourth-year students, respectively. Students, particularly fourth-year students, frequently noted that prior cold-run practice helped them understand the procedural steps and approach hot-run training with a greater sense of preparedness.

#### Learning impressions and perceived retention

Statements related to learning impact, strong impressions, and retention were identified in 50.3% of the responses (72 responses), including 43 from third-year students (53.8%) and 29 from fourth-year students (46.0%). Many students reported that repeated hands-on experience and the tension associated with real procedures contributed to stronger impressions and perceived memorability. Conversely, a subset of responses expressed concerns about reproducing the same procedures after time had elapsed or performing them independently, indicating uncertainty regarding sustained confidence in performing the procedures.

#### Perceived usefulness in future clinical practice

Comments indicating that training would be useful for future clinical practice accounted for 21.0% of the responses (*n* = 30). This theme was observed more frequently among third-year students (23 responses, 28.8%) than among fourth-year students (seven responses, 11.1%). Students noted that experiencing unsealed radioactive source handling during training helped them envision clinical practice, although this perception was reported less frequently by fourth-year students.

## Discussion

### Overview of the main findings

This study explored students’ subjective educational experiences associated with cold- and hot-run training involving unsealed radioactive sources. Overall, hot-run training was associated with higher perceptions of safety awareness, clinical realism, and learning satisfaction, whereas cold-run training supported procedural preparation and foundational understanding. In contrast, items reflecting higher learning stages, such as confidence in independent performance (A4, B4, and C4) and the ability to teach others (A5, B5, and C5), demonstrated relatively lower scores, indicating a gap between perceived experiential understanding and self-confidence in autonomous performance, which is consistent with the learning pyramid concept [13, 14]. In the present study, similar tendencies were observed in which the subjective ratings related to procedural understanding and safety awareness were relatively high, whereas confidence in independent performance and teaching others remained comparatively low.

These findings suggest that cold- and hot-run training may contribute to different dimensions of students’ learning experiences and should therefore be interpreted as complementary rather than competing educational approaches. Previous studies on experiential and simulation-based healthcare education have similarly suggested that preparatory simulation may improve learner preparedness and procedural familiarity before exposure to real clinical environments [15], whereas simulation-based training may enhance learners’ confidence and perceived readiness for clinical practice [16]. These findings align with students’ perceptions in the present study.

### Experiential learning and clinical realism

Qualitative analysis revealed educational characteristics associated with hot-run training. Descriptions involving tension, fear, and perceived danger accounted for 39.2% of the responses, and were observed across both academic years. These emotional and experiential elements were rarely mentioned in cold-run training and were not fully captured by Likert-scale evaluations, highlighting the complementary value of qualitative analysis.

Additionally, hot-run training was associated with considerably higher scores for self-reported understanding of radiopharmaceutical handling (A1), perceived procedural understanding (A2), and surface contamination measurements (A3). These findings suggest that experiential exposure to real radioactive materials may contribute to students’ perceptions of clinical realism and procedural engagement. Previous studies on experiential learning and simulation-based healthcare education have suggested that realistic clinical contexts and immersive practical experiences enhance learner engagement and reflective understanding during practical training [17–19]. Lateef [17] emphasized that realistic simulation environments facilitate experiential learning by reproducing the emotional and situational aspects of clinical practice, whereas Issenberg et al. [18] reported that realism and active participation are important components of effective simulation-based learning. Cant and Cooper [19] also showed that simulation-based education may enhance learner engagement and perceived preparedness for healthcare training.

Our findings corroborate these previous studies. Additionally, qualitative analysis demonstrated that students frequently perceived tension, fear, realism, and heightened awareness during the hot-run training. These experiential and emotional characteristics were not sufficiently reflected by the Likert scale scores alone, suggesting that the qualitative analysis contributed to a more detailed understanding of the subjective educational experiences associated with handling unsealed radioactive sources.

### Safety awareness and radiation risk perception

Safety-related items, including awareness of safe behaviors (C1), exposure reduction (C2), and contamination prevention (C3), were significantly higher in the hot-run training group. Both quantitative and qualitative findings suggest that heightened perceptions of safety management and radiation risk awareness are associated with hot-run training. Frequent qualitative references to responsibility and exposure control suggest that students experienced radiation safety awareness more concretely during hot-run training, rather than perceiving it merely as abstract theoretical knowledge.

Furthermore, among fourth-year students, a significant difference between the cold- and hot-run training persisted only in terms of safety awareness (C3). Although the present cross-sectional study design does not allow a longitudinal interpretation, this finding suggests that experiential exposure to unsealed radioactive sources is more strongly associated with sustained perceptions of safety awareness than procedural understanding or self-confidence. Previous studies have reported that experiential and simulation-based learning may contribute to the retention of confidence, preparedness, and safety-related awareness in healthcare education [20, 21]. Zieber and Sedgewick [20] demonstrated that experiential learning experiences are associated with sustained confidence and knowledge retention among nursing students, whereas Kochhar et al. [21] reported that practical and immersive training experiences contribute to long-term retention following basic life support education.

Our results align with these reports. In addition, the qualitative findings of the present study suggest that students not only conceptually understood radiation safety but also perceived responsibility, exposure control, and contamination prevention more concretely during hot-run training. These findings suggest that experiential exposure to real radioactive materials may contribute to the subjective recognition of radiation safety in ways that are not fully captured by quantitative scores alone.

### Complementary roles of cold- and hot-run training

Qualitative responses indicated that cold-run training was perceived as supporting procedural understanding and reducing the psychological burden during hot-run training. Items without significant differences between runs suggest that cold-run training may have provided a foundational learning experience. Therefore, these two approaches should be interpreted as complementary educational methods rather than substitute training approaches.

As studies directly comparing cold- and hot-run training in nuclear medicine education remain limited, the present findings were interpreted with reference to healthcare education literature comparing simulation-based preparatory learning with realistic clinical exposure [22, 23]. McGaghie et al. [22] emphasized that simulation-based education functions as preparatory learning to facilitate the transition to practical clinical situations, whereas Okuda et al. [23] reported that simulation-based learning provides a safe environment for experiential preparation before actual clinical exposure. Within this framework, cold-run training may correspond to simulation-based preparatory learning, whereas hot-run training may reflect experiential learning in realistic clinical environments. The present findings corroborate previous reports and suggest that students subjectively perceive cold- and hot-run training as educational approaches with different but complementary roles.

### Educational implications and future simulation approaches

Despite strong short-term subjective impressions, concerns regarding the retention of procedural understanding and confidence in independent performance were observed, consistent with the lower scores observed in the fourth-year students. Considering the practical limitations associated with repeated hot-run training, including radiation safety management and institutional constraints, alternative simulation-based educational approaches may have practical value.

Recent studies have explored virtual reality (VR), augmented reality (AR), and simulation-based approaches for radiation safety and radiological education. Guo et al. [24] developed a VR-based radiation safety training system for interventional radiologists. Nishi et al. [25] reported that AR/VR educational materials can visualize scattered radiation behavior, supporting learners’ understanding of radiation protection. Rainford et al. [26] further showed that 3-D VR simulation was perceived positively by radiography and medical students during radiation protection training. In addition, Leek et al. [27] suggested that nonimmersive VR simulations may support a conceptual understanding of radiation and risk.

In nuclear medicine education, Marshall et al. [28] reported the use of immersive VR for radiologically hot laboratory simulations, and Kakimoto et al. [29] demonstrated the educational usefulness of VR-based training for radiopharmaceutical administration. These studies suggest that VR-based approaches may provide safe, repeatable, and visually intuitive learning environments for procedures that are difficult to practice repeatedly in radiation-controlled settings.

The present study suggests that cold- and hot-run training contribute to different dimensions of students’ learning experiences. Considering the practical limitations associated with repeated hot-run training, previous studies on VR- and simulation-based education [24–29] suggest that immersive and repeatable training environments may provide additional opportunities for experiential reinforcement under safe and controlled conditions.

### Study limitations

This study has several limitations, including its cross-sectional and single-institution design. Moreover, comparisons between cold- and hot-run training were based on students’ subjective perceptions rather than a randomized controlled experimental framework. Furthermore, owing to the cross-sectional design, comparisons between third- and fourth-year students reflect differences between independent cohorts assessed at different post-training intervals rather than longitudinal within-subject changes. Differences in assessment timing may have also introduced a recall bias related to memory and retrospective evaluation.

In addition, this study aimed to evaluate students’ subjective educational experiences and perceptions associated with cold- and hot-run training rather than objective clinical performance or competency acquisition. Therefore, these findings should be interpreted as subjective indicators based on self-reported evaluations. Objective performance-based assessments, such as OSCE-based or structured practical performance evaluations, were not performed in this study.

Finally, although the qualitative analysis was conducted using independent review procedures, the researchers’ subjective interpretations may have influenced the processes of category classification and theme extraction. Future studies incorporating longitudinal designs and objective performance-based assessments are warranted.

## Conclusions

This study provides a structured quantitative evaluation of hot- and cold-run perceived safety-related learning experiences, using a structured assessment scale for nuclear medicine education. It identified distinct educational characteristics associated with hot-run practical training using unsealed radioactive sources, which have not been clearly established in previous studies. Hot-run training was associated with higher levels of perceived safety awareness and experiential learning experiences than cold-run training, supporting its complementary role in radiological technologist education. 

## Supplementary Information

Supplementary Material 1.

## Data Availability

The datasets generated and analyzed during the current study contain sensitive information from human participants and are not publicly available to protect participant privacy. Access to the data may be granted upon reasonable request to the corresponding author, subject to approval by the institutional ethics committee and compliance with ethical and privacy regulations.
