# Supplementary material for: Educational experiences associated with cold- and hot-run practical training on the handling of unsealed radioactive sources by radiological technologist students
Source: BMC Med Educ. 2026 Jun 5;26:1301. doi: 10.1186/s12909-026-09594-w (PMC13465457; doi:10.1186/s12909-026-09594-w)
Supplement: Supplementary file 1 [file 12909_2026_9594_MOESM1_ESM.pdf]

Supplemental File 1

Primary outcomes	Secondary outcomes	1 = strongly disagree	2 = disagree	3 = neutral	4 = agree	5 = strongly agree
A. Procedural understanding & confidence	A1. Understood the procedures for direct measurement	☐	☐	☐	☐	☐
(Surface contamination measurement)	A2. Understood the procedures for indirect measurement	☐	☐	☐	☐	☐
	A3. Understood the calculation methods and criteria for removal from controlled areas	☐	☐	☐	☐	☐
	A4. Confident in performing all three procedures	☐	☐	☐	☐	☐
	A5. Confident in explaining all three procedures to others	☐	☐	☐	☐	☐
B. Procedural understanding & confidence	B1. Understood the operational procedures for the generator	☐	☐	☐	☐	☐
(Syringe/vial handling, ^{99m}Tc generator operation)	B2. Understood the operational procedures for radiopharmaceutical dispensing	☐	☐	☐	☐	☐
	B3. Understood calculations for generator, radioactive decay, and decay correction	☐	☐	☐	☐	☐
	B4. Confident in performing all three procedures independently	☐	☐	☐	☐	☐
	B5. Confident in explaining all three procedures to others	☐	☐	☐	☐	☐
	C1. Understood safety procedures for handling unsealed radioactive sources	☐	☐	☐	☐	☐
	C2. Learned practical methods to reduce radiation exposure	☐	☐	☐	☐	☐
C. Safety awareness & Radiation protection	C3. Felt that safety awareness increased through this training/operation	☐	☐	☐	☐	☐
	C4. Confident in performing all three items independently	☐	☐	☐	☐	☐
	C5. Confident in explaining all three items to others	☐	☐	☐	☐	☐
	D1. Felt that the learning effects through the training and procedures were sufficient	☐	☐	☐	☐	☐
D. Learning Satisfaction	D2. Satisfied with the content and operations of this training	☐	☐	☐	☐	☐
	D3. Felt that this training/procedure will be useful for future work	☐	☐	☐	☐	☐
	E1. Felt confident in the knowledge and skills gained from this training/procedure	☐	☐	☐	☐	☐
E. Self-confidence & Self-assessment	E2. Felt capable of performing the same procedures independently	☐	☐	☐	☐	☐
	E3. Felt able to perform the procedures correctly in the future based on this experience	☐	☐	☐	☐	☐
Open-ended Response	-	Please describe any insights or learning experiences you gained from the practical training using unsealed radioactive sources.				
